# Efficacy and safety of “Jollab Monzej” as a traditional persian compound medicine for the treatment of multiple sclerosis-related fatigue: A randomized placebo-controlled trial

**DOI:** 10.22088/cjim.14.2.257

**Published:** 2023

**Authors:** Fatemeh Yousefnia Babaki, Mahmood Khodadoost, Hossein Rezaeizadeh, Abdorreza Naser Moghadasi, Shirin Fahimi, Hamed Hosseini, Mina Movahhed, Kurosh Gharagozli

**Affiliations:** 1 Department of Traditional Medicine, School of Traditional Medicine, Shahid Beheshti University of Medical Sciences, Tehran, Iran; 2 School of Persian Medicine, Tehran University of Medical Sciences, Tehran, Iran; 3 Multiple sclerosis Research Center, Neuroscience institute, Tehran University of Medical Sciences, Tehran, Iran; 4 Department of Traditional Pharmacy, Faculty of Pharmacy and Pharmaceutical Sciences, Tehran Medical Sciences, Islamic Azad University, Tehran, Iran; 5 Center for Research and Training in Skin Diseases and Leprosy (CRTSDL), Tehran University of Medical Sciences (TUMS), Tehran, Iran; 6 Brain mapping research Center, Loghman Hakim educational hospital, Shahid Beheshti University of Medical Sciences, Tehran, Iran

**Keywords:** Multiple sclerosis, Fatigue, Herb, Traditional, Persian Medicine

## Abstract

**Background:**

The purpose of this study was to investigate the efficacy and safety of Jollab monzej (JMZ), a Traditional Persian compound medicine, on multiple sclerosis-related fatigue (MSRF).

**Methods:**

We did a double-blind randomized controlled phase3 clinical trial on the JMZ syrup in fifty-six relapsing-remitting MS (RRMS) patients aged 18-55 years with moderate to severe fatigue using the Expanded Disability Status Scale (EDSS) score ≤ 6. We randomly assigned (1;1) participants to the JMZ syrup or placebo _syrup_ groups treated for one month. Participants, investigators, and assessors were unaware of the assignments. The primary outcome was changes in the fatigue score on the Fatigue Severity Scale (FSS), at baseline and one month after treatment using the intention-to-treat (ITT) analysis. The secondary outcomes were changes in the score of Visual Analogue Scale (VAS), Beck Depression Inventory (BDI), and Pittsburgh Sleep Quality Index (PSQI). Outcomes were measured at baseline, one month after treatment, and 2-week follow-up. Safety was detected in all participants.

**Results:**

We randomly assigned 56 participants to the JMZ group (n=28) and placebo group (n=28). Fatigue scores significantly changed in both groups; however, the JMZ group had a greater reduction in FSS score in the ITT analysis. The adjusted mean difference was 8.80 (Confidence interval (CI) 95%, 2.90-14.70, P = 0.00). The mean difference of VAS, BDI, and global PSQI scores were statistically significant (P=0.01, P₌0.00, P₌0.01; respectively). Regarding safety, mild adverse events (AEs) were reported.

**Conclusion:**

The results of our study revealed that the administration of JMZ syrup alleviated MSRF and also could improve depression and sleep disorders.

Multiple sclerosis (MS) is a chronic immune-mediated disease of the central nervous system (1). The prevalence and incidence of MS in Iran is high and is increasing over the time (2). Fatigue is one of the most common and debilitating symptoms in MS patients, which greatly affects the patients’ quality of life, work performance, and social interactions (3). The prevalence rate of MS-related fatigue (MSRF) has been estimated to be 52-88% in various studies (4). The MS Council for Clinical Practice Guidelines has described fatigue as a subjective lack of physical and/or mental energy that is perceived by the individual or caregiver to interfere with usual and desired activities (3). 

There is no definitive treatment for this disorder. Several pharmacological and non-pharmacological interventions have been applied for the treatment of MSRF. Pharmacological treatments are including amantadine, modafinil, aspirin, acetyl-L-carnitine, pemoline, and 4-aminopyridine and also herbal treatments such as Ginseng and Ginkgobiloba are used in MS fatigue (5,6). Both peripheral (e.g. muscular fatigue) and central mechanisms are involved in the pathophysiology of MSRF, although evidences demonstrate that central mechanisms are the most important ones, in MSRF. Secondary fatigue is a consequence of other comorbidities that may be related to MS or other disease, such as sleep and mood disorders (7,8). Because of The multifactorial nature of MSRF and our incomplete understanding of MSRF pathogenesis, its management is a challenge and the multidisciplinary approach seems to be the best solution (3,9). 

The integrative and complementary health approach (ICHA) is frequently used by patients with serious physical or mental diseases (10). MS patients commonly use complementary and alternative medicine (CAM) to treat illness or relieve their symptoms. Cross-sectional studies showed that 37%–100% of MS patients have ever used CAM and up to 51.8% of them have used it in the past 12 months (11,12). 

In this regard, traditional herbal remedies can be appraised for alleviating the symptoms of MS. A few clinical trials have been performed to evaluate the effectiveness of Persian Medicine (PM) formulations on MSRF. In our study the Jollab monzej (JMZ) syrup that is composed of Jollab, a mixture of saffron with rose water, and the aqueous extract of lemon balm, Lavander and Iranian borage was specified to evaluate in MSRF. Various effects of this herbs including antioxidant, anti-inflammation, neuroprotective, hypnotic, antidepressant and anxiolytic properties, have been reported that seems to affect MSRF. In the present study, we aimed at assessing JMZ syrup, as a traditional medicine used in PM Pharmacopeias to investigate its safety and efficacy on MSRF. 

## Methods

**Trial design:** This study was a randomized, double-blind, parallel-group, placebo-controlled phase3 clinical trial, conducted in accordance with the Guidelines for Good Clinical Practice protocol and its amendments and the Declaration of Helsinki principles. The Ethical Scientific Committee of Shahid Beheshti University of Medical Sciences, Tehran, Iran, approved the study (NO.IR.SBMU.RETECH.REC.1397.1395NO), and also the trial was registered at the Iranian Registry of Clinical Trials (IRCTID: IRCT20190623043984N1). This study was reported according to the CONSORT guidelines (13). 

**Participants**: In our study, MS patients with a complaint of fatigue who were visited by academic neurologists of Shahid Beheshti and Tehran Universities were screened to evaluate eligibility criteria. This trial was carried out in the neurology outpatient clinic and office and also patients referred from the Iran MS Society were included. Inclusion criteria were as follows: patients aged 18 to 55 years with definite MS based on the McDonald criteria (14), clinical diagnosis of RRMS, an EDSS score of ≤ 6, a total score ≥ 36 on Fatigue Severity Scale (FSS), and signing the informed consent. Exclusion criteria were as follows: pregnancy and lactation, serious and major medical illness, clinically active infection, no relapse and no consumption of corticosteroides over the past 3 months before the study, uncontrolled psychiatric disorders that could affect treatment compliance, severe depression on Beck's Depression Inventory (BDI), and changing the disease-modifying drugs of MS in the last three months. Drop out criteria included the occurrence of relapse during the study, any side effects related to intervention resulted in non-compliance, changing the disease-modifying drugs during the study, poor adherence to treatment by not taking the medicine for at least three consecutive days, or irregular taking with a total dose of less than half the prescribed dose during a week, starting any medical treatments that can affect fatigue during the intervention period, and non-compliance.

**Intervention**: In this study, the JMZ syrup was used that is composed of Jollab, a mixture of saffron, rose water, and the aqueous extract of lemon balm, Lavander and Iranian borage. JMZ was prepared according to the documented Persian Medicine manuscripts (15,16). For this purpose, dried leave of *Melissa officinalis *L*.*, dried flowers of *Lavandula angustifolia Mill*. and dried petals of *Echium amoenum Fisch. & C.A. Mey.* were purchased from Tehran herbal market. Subsequent to identifying, herbal market samples (HMS) of *M. officinalis *L*.* (HMS No. 545)*, Lavandula angustifolia Mill.* (HMS No. 543) and *Echium amoenum Fisch. & C.A. Mey* (HMS No.544) were deposited at the Herbarium of Traditional Medicine and Materia Medica Research Center (TMRC), Shahid Beheshti University of Medical Sciences, Tehran, Iran. Rose water is the aromatic water that is obtained from the distillation of damask rose petals (*Rosa damascena Mill*.) and was purchased from Al-Zahra Company (Kerman, Iran, health certification code of 42/10947). Saffron (stigmas of *Crocus sativus* L.) was also purchased from Saharkhiz Company (Mashhad, Iran, health certification code 50/11500). In order to formulate JMZ, first, an aqueous extract of three plant materials (with equal proportions) was prepared using the decoction method (total plant materials: water, 1:13). By adding sugar (77% w/v), a suitable consistency was obtained for the herbal extract. The process was completed by adding pre-prepared Jollab (herbal extract: Jollab, 4:1). The Jollab was prepared using rose water (59.8%, w/v), sugar (40%, w/v), and saffron (0.2%, w/v). The final formulation was satisfactory concerning its physical parameters and microbial contents (17). Moreover, product standardization was performed based on total polyphenols of plant components (284 mg/100ml, based on pyrogallol) (18).

The placebo syrup was formulated with the same color and odor as JMZ; only the taste differed. Due to the false-positive effects, sugar was omitted from the placebo formulation. An edible licensed food coloring (powdered Caramel color 88K, MAPTRAL-15001D, MAP-DIANA Company, England,) was used so that the placebo resembled JMZ in color. Rose water (2%, w/v) and carboxymethyl cellulose (0.5%, w/v) were applied in placebo formulation to mimic the odor and consistency of JMZ, respectively. Both JMZ and placebo syrups were formulated and prepared in the Traditional Medicine and Materia Medica Research Center, Shahid Beheshti University of Medical Sciences, Tehran, Iran. The prepared syrups were stored in identical dark polyethylene terephthalate (PET) bottles labeled similarly. The patients took JMZ or placebo twice a day orally, each time 10 ml for weight < 60 kg and 15 ml for weight ≥ 60 kg, diluted in a cup of water before breakfast and at 5 p.m, for one month. The dosage of the syrup was determined considering the permitted range of components in pharmaceutical books and prescription orders and also a single-arm pilot study on MS patients (15,16,19,20).

**Outcome measures**: Fatigue changes as the primary outcome were measured using the FSS. The FSS is the most widely used fatigue scale with 9 items. Each item is scored from 1 (strongly disagree) to 7 (strongly agree), with a total score of 9 to 63. Higher scores point out more severe fatigue (21,22). The secondary outcome was changes in the score on the Visual Analogue Scale (VAS), BDI, and global score of the Pittsburgh Sleep Quality Index (PSQI). VAS is used for the overall subjective feeling of fatigue and its score ranges from 0 to 10, with lower scores indicating a better condition. Depression was assessed by the BDI with a total score between 0 and 63 and also the scores > 30 indicate severe depression that was considered in exclusion criteria (23,24). For evaluation of the sleep quality, PSQI with seven subscales was used. The total score on this scale is the sum of the scores of the subscales, ranging from 0-21, with a score ≥ 5 indicating poor sleep quality (25). Questionnaires were completed under the guidance of an investigator, at baseline, one montgh after treatment, and two-weeks follow-up. Furthermore, the valid and reliable Persian version of the questionnaires was applied to participants (22,23,25).

**Safety: **In our study, a self-report form was given to participants to report any adverse events (AEs) during study period along with the phone number of the investigator to tell their problems and emergency AEs. In addition, the investigator’s findings obtained from making phone calls or in-person visits of the subjects were recorded to evaluate AEs.

**Sample size: **The minimum important difference in fatigue score on FSS, which is clinically significance in MS, is estimated to be 0.45 to 0.88 (26). Using a two-sided two-sample equal-variance t-test, at least 42 participants were needed to achieve the population mean difference 4.5 points in FSS total score with a significance level (alpha) of 0.050 to reject the null hypothesis of equal means and 80% power and also a standard deviation of 5 for both groups. Considering the loss of 20% of the subjects, finally, 28 participants were calculated in each group. 

**Randomization, concealment, and blinding: **In our study, permuted block randomization with a block size of 4, generated by a computer, was carried out. Each random sequence was matched with a code containing two letters and one number. The code was used for enrollment so that the participants were not informed about their group assignment. In addition, the prepared syrups were stored in identical dark polyethylene terephthalate (PET) bottles with a similar label so that they could not be identified by their appearance. In this trial, an investigator who carried out enrollment of the participant, treatment process, and data collection, was different from specialists who treated patients. Therefore, these persons and a researcher who assessed the outcomes were blinded to group allocation until the end of the study.

**Statistical methods: **Continuous and categorical variables were demonstrated with mean ± standard deviation (SD) and frequency/percentage, respectively. Intention-to-treat (ITT) analysis was used to detect the efficacy of outcome measures. T-test and Chi-square test were used to compare the demographic characteristics of the two intervention groups. A repeated measure analysis of variance (ANOVA) adjusted with baseline scores, was used to detect differences between the groups at baseline, one month after treatment, and two-weeks follow-up. Statistical tests were two-tailed. A 95 % confidence interval was calculated and a p-value < 0.05 was considered as the significance level. 

## Results

**Figure 1:**
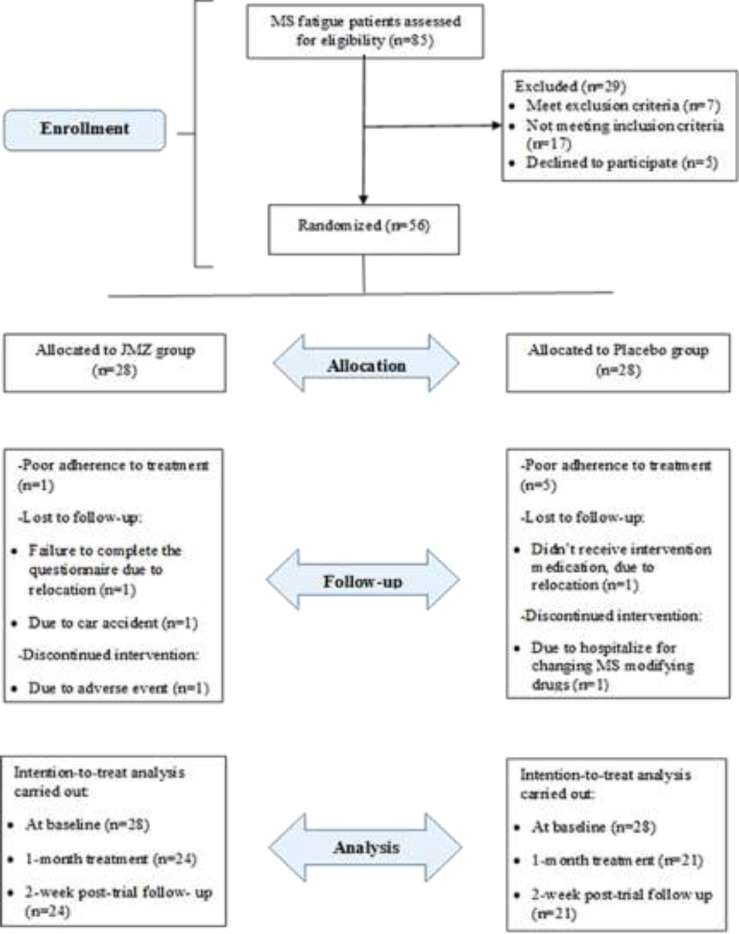
CONSORT flow diagram

**Recruitment**: From January to September 2020, a total of 85 MS patients with fatigue complaints were screened and assessed for eligibility criteria. We randomly assigned (1;1) participants to the JMZ group (n=28) or placebo group (n=28) treated for one month, so that 56 randomized patients entered into the ITT population analysis. During the treatment period, 11 participants dropped out, including 7 subjects in the placebo group and 4 subjects in the JMZ group (Figure 1). Baseline demographic and clinical characteristics of the subjects in the two groups are shown in table 1. There were no significant differences at baseline between the two groups except for the mean time (year) since diagnosis that was significantly longer in the JMZ group than the placebo group (P =0.02).

**Outcomes: **Our study results showed the reduction in FSS score from the baseline was significantly greater in the JMZ group and the adjusted mean score difference was 8.80 (95% CI, 2.90-14.70, P = 0.00). In addition, compared to the baseline, the mean score reduction in the VAS was significantly greater in the JMZ group than the placebo group (95% CI, estimated mean difference: 1.24, 0.25-2.22, P = 0.01).

 The mean BDI score reduction from baseline was significantly greater in the JMZ group than the placebo group (95% CI, estimated mean difference: 5.21, 2.25-8.17, P = 0.00). Furthermore, the mean PSQI global score changed significantly in the JMZ group than the placebo group (95% CI, estimated mean difference: 1.84, 0.38-3.29, P = 0.01). The mean changes of variables was reported in table 2.

**Safety assessment results** **: **JMZ was well tolerated in patients. No severe AEs were reported throughout the study period. Eleven subjects in the JMZ group and six subjects in the placebo group reported one or more problems. The most common AEs during the treatment period were localized skin rashes and increased appetite with weight gain < 2 kg. Two patients reported increased appetite who had decreased appetite previously; therefore, they were satisfied with this and also with weight gain. Skin lesions appeared as localized red papule rashes or exacerbation of previous eczema on the abdomen and face. Other less common AEs possibly related to the study were reported in table 3. All AEs ameliorated with an intervention, such as lowering the dose or changing the time of taking the medicine except skin rashes and increased appetite, which continued for a while. Overall, the severity of possible AEs was mild and only one patient discontinued treatment because of AEs, including skin rashes and PND (Table 3). 

**Table 1:** Baseline demographic and clinical characteristics

**Variables**	**JMZ group** **(n=28)**	**Placebo group** **(n=28)**	**P-value**
**Age (year)** Mean [SD] Min, Max	38.17 [8.26] 23, 55	37.82 [8.47] 22, 55	0.87
**Sex, No. (%)** Female Male	24 (82.8) 5 (17.2)	21 (75.0) 7 (25.0)	0.53
**Weight (kg)** Mean [SD] Min, Max	68.28 [15.77] 52, 115	71.37 [12.04] 52, 100	0.41
**Education, No. (%)** ≤ 12 years 13-16 years > 16 years	8 (27.6) 16 (55.2) 5 (17.2)	14 (50) 7 (25) 7 (25)	0.85
**Years since diagnosis** Mean [SD] Min, Max	10.34 [7.07] 1, 30	6.67 [4.88] 1, 19	0.02*
**Disease-modifying therapies (DMTs), No. (%)** First-line Second-line Third-line Other	14 (48.3) 9 (31.0) 4 (13.8) 2 (6.9)	10 (35.7) 6 (21.4) 6 (21.4) 6 (21.4)	0.30

JMZ: Jollab monzej; SD: standard deviation

**Table 2:** Mean changes of dependent variables during the study period

**Variable** **Mean, [SD]**	**Baseline**		**1-month treatment**		**2-week follow-up**		**P-value**
	**JMZ**	**Placebo**	**JMZ**	**Placebo**	**JMZ**	**Placebo**	
**FSS,**	43.93 [7.98]	49.60 [8.06]	32.75 [11.89]	46.39 [12.92]	34.96 [12.40]	46.21 [12.72]	0.00*
**VAS,**	6.58 [1.57]	7.14 [1.86]	4.58 [2.38]	6.25 [2.06]	5.13 [2.34]	6.28 [2.01]	0.01
**BDI,**	13.55 [6.65]	18.14 [8.90]	7.06 [5.29]	15.53 [9.52]	8.96 [7.16]	16.17 [9.63]	0.00
**PSQI,**	8.55 [3.73]	8.64 [4.80]	4.83 [3.72]	6.57 [2.07]	6.24 [3.35]	7.78 [4.66]	0.01

* Repeated measures ANOVA; JMZ: Jollab monzej; FSS: Fatigue Severity Scale; VAS: Visual Analogue Scale; BDI: Beck Depression Inventory; PSQI: Pittsburgh Sleep Quality Index

**Table 3:** Possible Adverse events in the subjects

**Possible related-adverse events** **No. (%)**	**Placebo group** **(n** **₌** **28)**	**JMZ group** **(n** **₌** **28)**
**Skin lesions**	0 (0)	4 (14.28)
**Increased appetite with weight gain < 2Kg**	1 (3.57)	4 (14.28)
**Reduced appetite with weight loss ≤ 1Kg**	1 (3.57)	1 (3.57)
**Delayed bedtime**	1 (3.57)	2 (7.14)
**Daytime sleepiness**	0 (0)	1 (3.57)
**Headache**	1 (3.57)	1 (3.57)
**Sore throat**	0 (0)	1 (3.57)
**Nausea**	0 (0)	1 (3.57)
**Bloating**	1 (3.57)	0 (0)
**Epigastric pain**	2 (7.1)	1 (3.57)
**Anxiety**	0 (0)	1 (3.57)
**Post-nasal drip**	0 (0)	1 (3.57)

## Discussion

The aim of this controlled trial was to investigate the efficacy and safety of JMZ, a Persian herbal formulation, on moderate to severe MSRF. Our results showed an improvement in fatigue score on FSS in the JMZ group compared with the placebo group, indicating that the JMZ formulation was effective on MSRF. Furthermore, changes in the FSS score reached the minimal clinically important changes of MS fatigue (26). Fatigue is the most common problem in MS patients and there is no definitive treatment for this disorder. Several pharmacological and non-pharmacological interventions have been applied for the treatment of MSRF. A systematic review was conducted in 2016 on clinical trials on medications used in MSRF, including amantadine, modafinil, aspirin, acetyl-L-carnitine, pemoline, and 4-aminopyridine. The results of this showed that amantadine might be the only medicine with sufficient evidence to improve MSRF and modafinil was not significantly beneficial. These drugs except pemoline were tolerated well and the main AEs were gastrointestinal problems and insomnia. However, a few studies have been conducted in this field and data are limited. In addition, as the used sample sizes are relatively small; hence, high-quality RCTs are suggested (5).

According to medicinal herbs' effectiveness, a few clinical trial studies were carried out in MSRF. Etemadifar et al. performed a trial on ginseng effects administrated for three months in 52 patients with MS fatigue using the Modified Fatigue Impact Scale (MFIS) and their results showed a significant improvement in patients; however, a pilot study by Kim et al. on the effect of ginseng administrated for 6 weeks showed no improvement in MSRF (27). Another study on the effect of high- or low-flavonoid cocoa in 40 MS patients indicated no significant effects on MSRF and also moderate effect on fatigability (28). Evening primrose oil was reported to improve MSRF and quality of life in 52 subjects (29). Two traditional formulations were investigated on MS fatigue by Adalat et al. and Namjooian et al. in 52 and 60 patients, respectively, and their results showed significant improvement in MS fatigue (30,31). The results of the secondary outcome in the present study showed that changes in the VAS, BDI, and PSQI scores were significant between the two groups, suggesting that the JMZ syrup might be beneficial for depression and sleep disorders in MS patients.

Overall, there are some strong points in our study. Firstly, the JMZ syrup with a relatively good taste and the odor was consumed by the participants like a drink considering the taste is especially important about syrups. Secondly, one of the most common AEs was insomnia in mentioned trials. Our study showed that the JMZ syrup improved significantly PSQI global score between groups. Finally, the score ≥ 36 was considered as a cut-off point for FSS as an eligible criterion, which specifies MSRF so that the efficacy of JMZ was evaluated on moderate to severe MSRF, whereas in some studies, it has not been considered. Since JMZ formulation was not similar to other herbal formulations, it is not possible to compare the results with other studies. Therefore, constituents of JMZ syrup were evaluated as follows:

JMZ syrup contains herbs, including *C. sativus* L., *R. damascena Mill*., and *M. officinalis *L., *L. angustifolia Mill. and Echium amoenum Fisch. & C.A. Mey. *(Iranian Borage) which the effectiveness of their bioactive components have been demonstrated in various studies. Mizuma et al. showed that daily administration of crocetin (15 mg; a component of *C. sativus* L.) significantly reduced physical fatigue in men compared to the ascorbic acid and placebo (32). Moreover, crocetin has been suggested as an oxygen diffusion enhancer in both in vivo and in vitro studies (33). Osama et al. in an animal study found that distilled water of *R. damascene* flowers improved Hgb concentration and red blood cells count, which may be due to its anti-oxidant property (34). Some of these herbs have been shown to affect the immune system (*R. damascena*, *M. officinalis* and Iranian Borage) (34–36). Also, several activities of these herbs on the nervous system were reported, including their neuroprotective (*C. sativus* L., *M.* *officinalis* L. and *L. angustifolia Mill*.) and neurogenesis (*R. damascena* MILL.) properties (37–40). They can also improve cognition and memory function (Iranian Borage, L. *angustifolia Mill., R. damascena* L.) (40–42). In addition, these plants have been reported with anti-depressant, anti-anxiolytic effects and improvement in sleep quality (43–46). Antioxidant and anti-inflammatory properties of their bioactive metabolites have also been identified in different studies. It seems that herbal constituents of JMZ formulation can affect MSRF through multiple properties reported in different mentioned studies.

All these plants were reviewed in relevant studies, in which their several properties have been mentioned in PM texts including they were found as strengtheners of the brain, nerve, and other organs. They could enhance the vigor of the body and facilitate the removal of waste materials in the body. Antidepressant, sedative, and hypnotic effects were other properties in these plants (47–49). Regarding safety, mild AEs were reported and except for one patient in the JMZ group, other subjects with AEs continued the treatment period. The most common reported AEs were localized skin rashes and increased appetite with a weight gain of < 2 Kg. Possible related-AEs are listed in Table 3. Our study had some limitations. The intervention period was relatively short. Regarding the placebo, similarity to the JMZ syrup in taste and odor was important. The color of both was similar; therefore, there was no problem in the appearance of them and the smell of placebo syrup due to the presence of rose water was comparable with herbal syrups for participants, however their taste were different. The traditional Persian compound medicine JMZ syrup was well tolerated and alleviated fatigue in MS patients and also could improve depression and sleep disorders.
